# Drug-Induced Lupus with Leukocytoclastic Vasculitis Associated with Apixaban

**DOI:** 10.1155/2021/8866761

**Published:** 2021-02-13

**Authors:** John Yang, Steffi Lena, Daniel Tarditi, Gopika Banker

**Affiliations:** ^1^Jefferson Health, Philadelphia, PA, USA; ^2^The Heart House, Washington Township, NJ, USA; ^3^Nephrology and Hypertension Associates of NJ, Voorhees, NJ, USA

## Abstract

Drug-induced lupus is an iatrogenic-induced autoimmune disease with common offending agents well documented in the literature. To our knowledge, there are no prior case reports of drug-induced lupus associated with apixaban or any other direct oral anticoagulant. We describe a case of drug-induced lupus with leukocytoclastic vasculitis associated with apixaban started 15 days prior, after a WATCHMAN procedure for atrial fibrillation in an 86-year-old male previously anticoagulated on rivaroxaban.

## 1. Introduction

Drug-induced lupus (DIL) is an iatrogenic-induced autoimmune disease that can present systemically or cutaneously and varies in presentation based on the offending drug. Common systemic features include arthralgia and myalgia, while cutaneous lesions generally involving the lower extremity can include purpura or necrotizing vasculitis [1]. Leukocytoclastic vasculitis (LCV) is a histopathologic diagnosis with immune complex deposition into the vessel walls. Although LCV can be systemic, it most commonly is limited to cutaneous manifestations with palpable purpura in the lower extremities at 7–14 days after exposure to drug or infection [2]. DIL and LCV are not commonly described together nor commonly associated with apixaban.

## 2. Method

### 2.1. Case Report and Review of Literature

#### 2.1.1. Case

**Figure 1:**
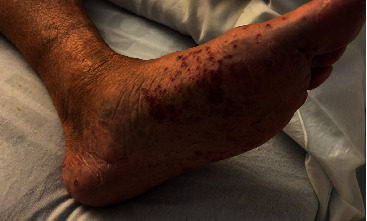
Rash of the left lower extremity.

An 86-year-old male presented with generalized weakness and dyspnea on exertion, additionally noted to have a new petechial rash on both of his feet without pruritus. The patient recently underwent placement of WATCHMAN left atrial appendage occlusion device and was converted from rivaroxaban to apixaban and aspirin, as well as from flecainide to amiodarone. Past medical history was significant for diverticulosis with partial bowel resection, paroxysmal atrial fibrillation complicated by multiple gastrointestinal bleeds, hypertension, type 2 diabetes mellitus, chronic kidney disease stage IIIb, major depressive disorder, and obstructive sleep apnea. Other medications include trazodone, citalopram, sitagliptin, metoprolol, pravastatin, oxybutynin, and tamsulosin. The patient denied chemical exposure, insect bites, or prior allergic reactions. On day 15 after starting apixaban, the patient noted nonpruritic petechial rash on his feet, followed by weakness and dyspnea on exertion leading to hospital admission (Figure 1). On physical exam, the patient had stable vitals on room air with an irregularly irregular rhythm, as well as a nonpruritic nonpainful nonblanching petechial rash beginning on the feet and later spread to his thighs and arms with associated diffuse myalgias. Laboratory data showed a white blood cell count of 11.3 with 0.3% eosinophil (0.3–5.0%), platelet count of 289, hemoglobin 12.1, creatinine 2.14 (baseline 1.5), erythrocyte sedimentation rate 63, C-reactive protein 11.7, borderline antinuclear antibody 1 : 40, strongly positive antihistone antibodies 4.4, positive hepatitis B core antibody, and C3 of 147 (75–152 mg/dL) with C4 of 72 (14–48 mg/dL). Further workup included negative double-stranded DNA, antineutrophil cytoplasmic antibodies, glomerular basement membrane antibodies, COVID-19, Rocky Mountain spotted fever, syphilis, coxsackie, and ehrlichia. A skin biopsy was performed. Apixaban was stopped, and the patient was started on prednisone 30 mg twice daily while waiting for skin biopsy results.

**Figure 2:**
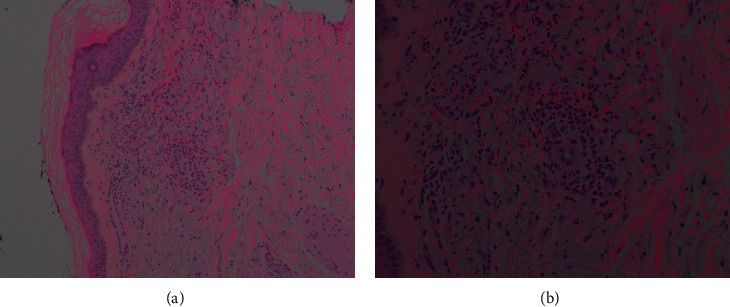
Excisional biopsy demonstrating superficial and deep perivascular neutrophil infiltration consistent with leukocytoclastic vasculitis.

Skin biopsy showed superficial and deep perivascular neutrophilic infiltration consistent with LCV (Figure 2). During the hospitalization, the patient was bridged onto warfarin with heparin and discharged with a prednisone taper. The rash and associated symptoms were resolved when seen at 2-week outpatient follow-up.

## 3. Discussion

Drug-induced lupus (DIL) is an iatrogenic-induced autoimmune disease that is typically suspected when the patient presents with systemic or cutaneous lupus-like symptoms in the context of a known DIL offender. None of the patient's existing or new medications are common offenders of DIL [3]. Combining the patient's cutaneous manifestations and recent change of medication, our initial clinical diagnosis was of apixaban-related leukocytoclastic vasculitis (LCV), of which there are four preceding case reports [4–7].

LCV is a small-vessel vasculitis with immune complex deposition into vessel walls with neutrophil activation, small vessel vasculature destruction, and red blood cell extravasation, resulting in palpable purpura preferentially seen in dependent areas, typically 7–14 days after initiation of the offending drug. When small vessel vasculitis is suspected, skin biopsy of fresh lesions within 24–48 hours should be obtained for histopathologic evaluation, looking for evidence of neutrophilic infiltrate of small vessels with evidence of granulocytic debris and nuclear dust, endothelial swelling, fibrinoid necrosis of vessel walls, and extravasation of red blood cells. Although skin biopsy is required for the diagnosis of LCV, positive findings do not rule out the possibility of extracutaneous involvement, and additional testing to exclude systemic involvement should be pursued. Management of LCV involves discontinuing the offending agent, with or without the addition of systemic immunosuppression. For severe cases involving widespread, ulcerative, or necrotic lesions, corticosteroids with a 4–8 week taper is appropriate [8].

As part of the workup to exclude systemic involvement, the rheumatologic panel revealed a strongly positive antihistone antibody titer [9]. After apixaban was discontinued and prednisone initiated, the symptoms dramatically improved without another likely causative agent. This supports that the patient had apixaban-related DIL. DIL typically presents with mild lupus-like symptoms including arthralgia, myalgia, fever, and cutaneous manifestations, with additional symptoms varying based on the offending drug. Skin lesions of note include photodistributed erythema and annular plaques, purpura, urticarial lesions, and necrotizing vasculitis. The management of DIL is similar to LCV, with discontinuing the offending drug, and initiation of corticosteroids for patients with end organ damage.

It is our impression that this patient had apixaban-related DIL with cutaneous manifestation consistent with LCV. To our knowledge, this is the first case report of DIL associated with a direct oral anticoagulant. This is the fifth case report of LCV associated with apixaban. There is one other case report of DIL with LCV, and it is documented in association with adalimumab [10].

## 4. Conclusion

Apixaban is a potential precipitant of drug-induced lupus that was not previously described in the literature. While leukocytoclastic vasculitis may have a similar cutaneous presentation, it is important to remember that it is a diagnosis of exclusion and to keep a wide differential. Fortunately, the general treatment direction for both diagnoses is similar, and in most cases, symptoms should improve and resolve after stopping the offending medication.

## Data Availability

The data used to support the findings of this study are available from the corresponding author upon request.
